# Supplementary material for: Investigating the quaternary structure of a homomultimeric catechol 1,2-dioxygenase: An integrative structural biology study
Source: PLoS One. 2025 May 5;20(5):e0315992. doi: 10.1371/journal.pone.0315992 (PMC12052123; doi:10.1371/journal.pone.0315992)
Supplement: S1 Table — (PDF) [file pone.0315992.s001.pdf]

S1 Table. List of buffers

Buffers Name	Buffers content	pH
Lysis Buffer	20 mM Tris-HCl<br>50 mM NaCl<br>10 mM Imidazole	8
Buffer B	20 mM Tris-HCl<br>50 mM NaCl	8
Buffer C	10 mM Tris-HCl	8
