# Supplementary figures and images for: Investigating the quaternary structure of a homomultimeric catechol 1,2-dioxygenase: An integrative structural biology study
Source: PLoS One. 2025 May 5;20(5):e0315992. doi: 10.1371/journal.pone.0315992 (PMC12052123; doi:10.1371/journal.pone.0315992)

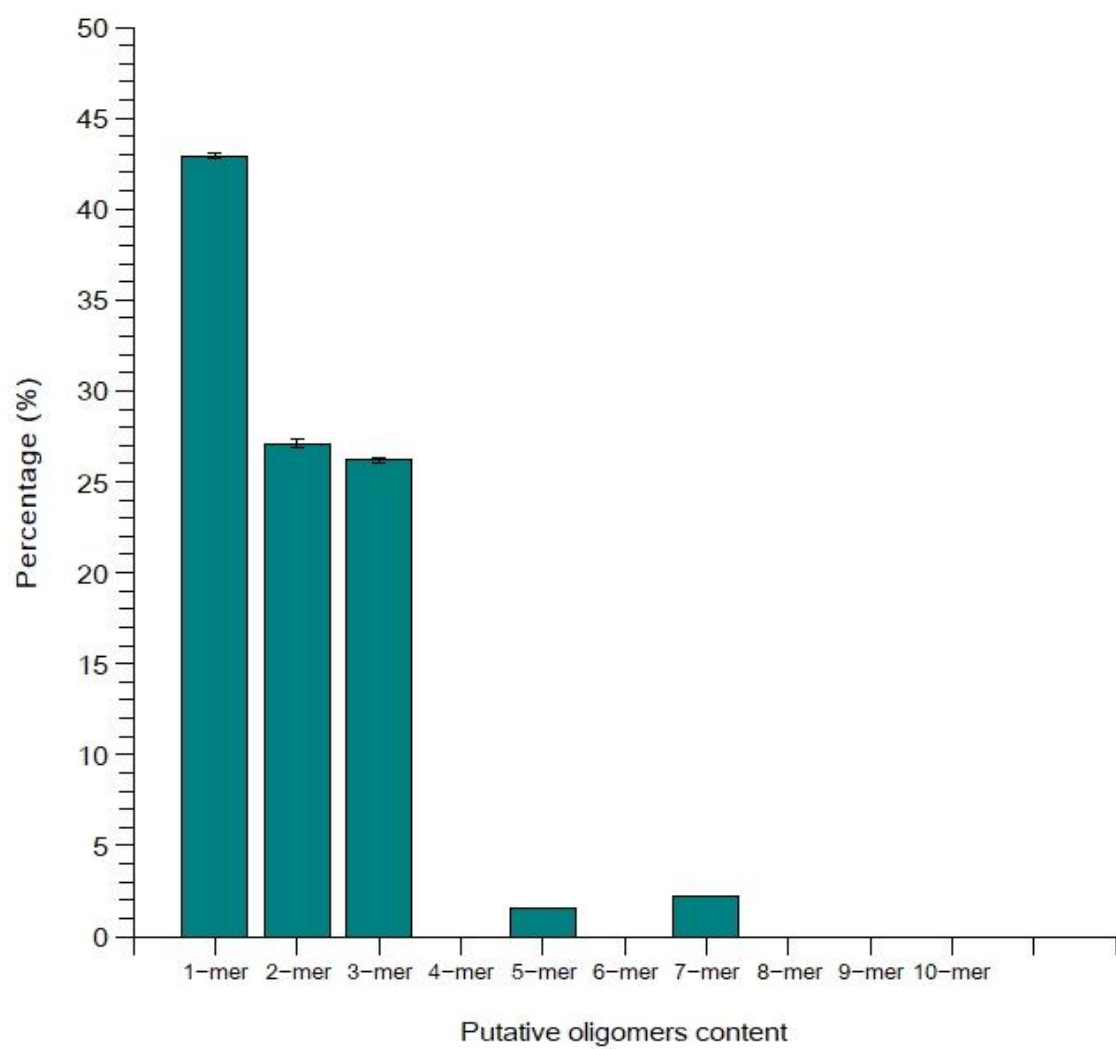

Supplement: S2 Fig — The graph shows the identified oligomers in the % content of SfC12DO lyophilized samples. These oligomeric forms were identified using the OLIGOMERS tool from ATSAS. (PDF) [file pone.0315992.s002.pdf]

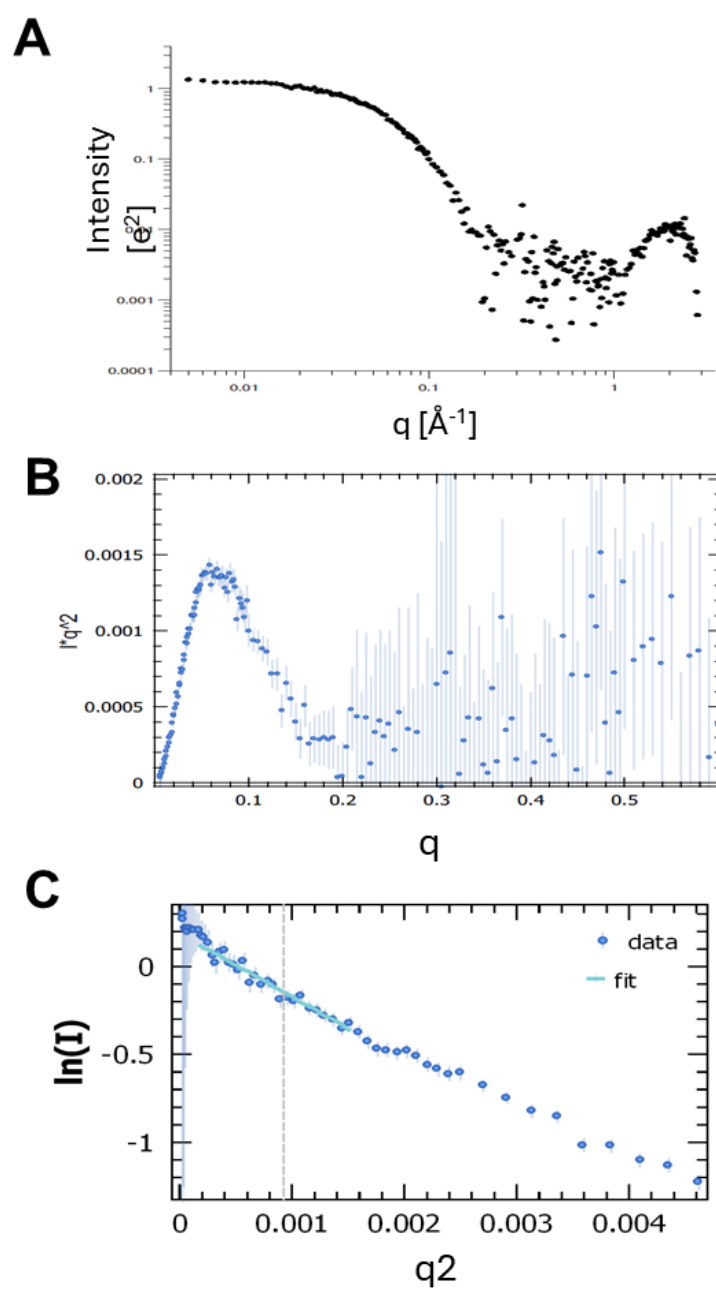

Supplement: S3 Fig — (A) The SAXS intensity profile is presented as a function of the scattering vector q [Å ⁻ ¹]. (B) The Kratky plot, which shows q²I(q) against q, reveals the absence of a well-defined peak and a gradual increase at higher q values. This suggests that non-lyophilized SfC12DO experiences partial disorder or unfolding. (C) The Guinier plot, depicting ln(I) versus q², further indicates that non-lyophilized SfC12DO exhibits characteristics consistent with a partially unfolded or flexible conformation. (PDF) [file pone.0315992.s003.pdf]

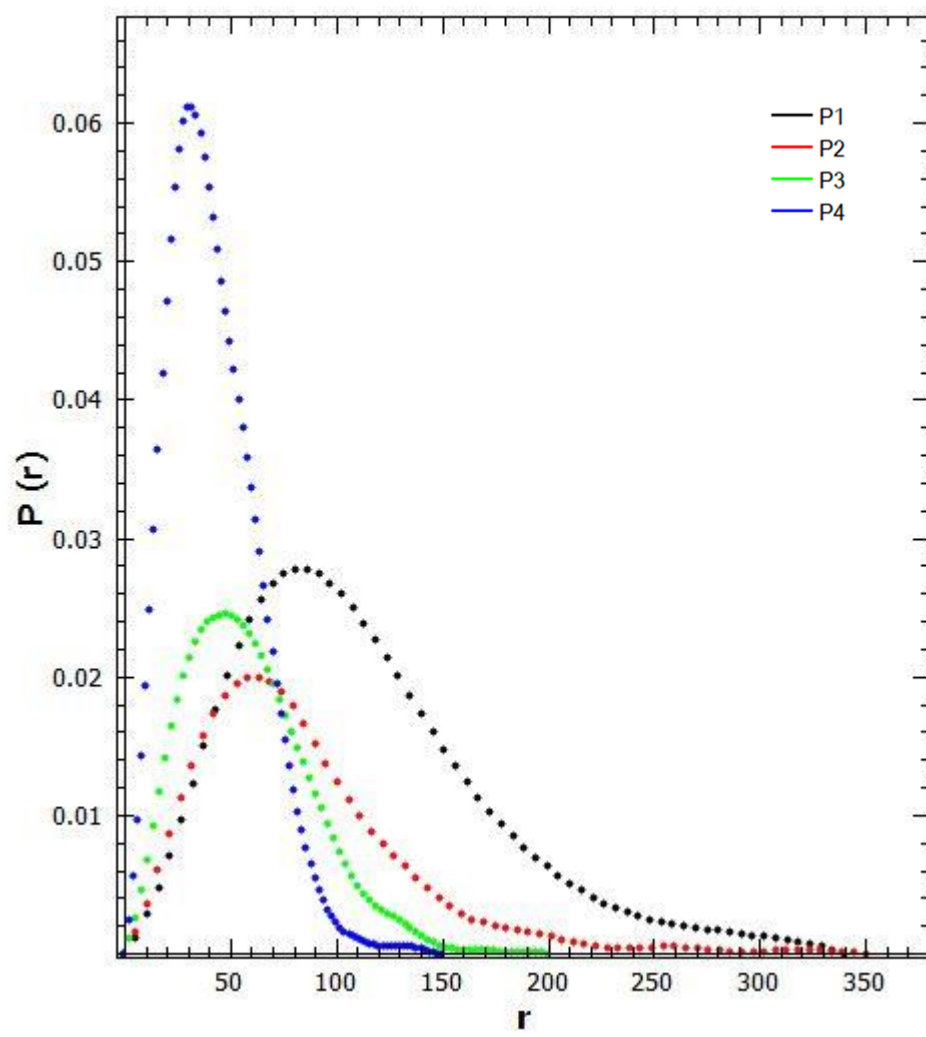

Supplement: S4 Fig — The curves are color-coded as follows: blue represents the dimer (p4), green denotes the tetramer (p3), red indicates the octamer (p2), and black corresponds to large aggregates (p1). The P(r) distributions exhibit distinct Rmax values, highlighting variations in size and shape among the oligomers. The distributions for the dimer and tetramer are well-defined and symmetric, while the octamer and large aggregates show broader profiles. This suggests that the latter two have increased structural complexity and potential flexibility. (PDF) [file pone.0315992.s004.pdf]

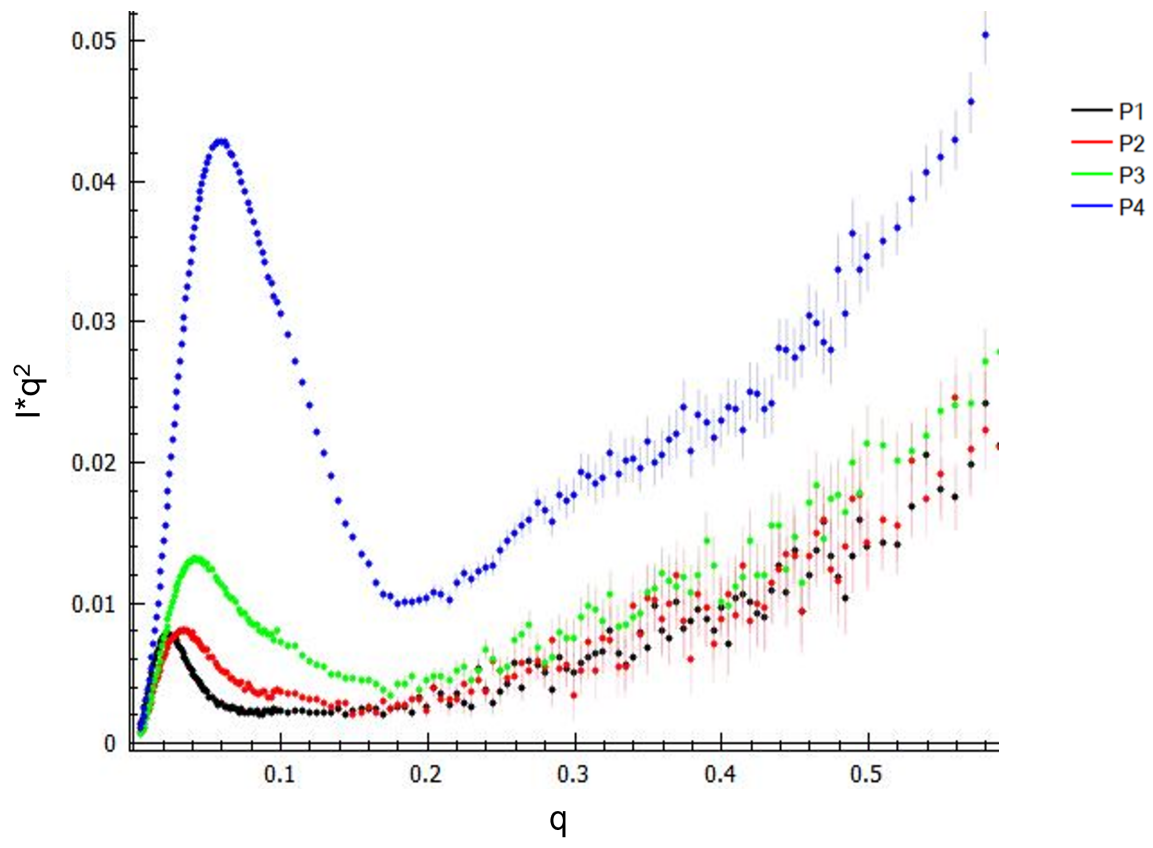

Supplement: S5 Fig — The curves indicate that the dimer (blue, p4) and tetramer (green, p3) display a more compact shape, while the octamer (red, p2) and larger aggregates (black, p1) show an increase at higher q values, suggesting a degree of flexibility. The variations in the profiles reflect differences in structural rigidity among the various oligomeric states. (PDF) [file pone.0315992.s005.pdf]
