# Supplementary material for: Investigating the quaternary structure of a homomultimeric catechol 1,2-dioxygenase: An integrative structural biology study
Source: PLoS One. 2025 May 5;20(5):e0315992. doi: 10.1371/journal.pone.0315992 (PMC12052123; doi:10.1371/journal.pone.0315992)
Supplement: Attachment — Submitted filename: Response to Reviewers.docx [file pone.0315992.s019.docx]

**PONE-D-24-55837**

**Investigating the quaternary structure of a homomultimeric catechol 1,2-dioxygenase: An integrative structural biology study.**

**PLOS ONE**

**Dear Dr. Rudiño-Piñera,**

**Thank you for submitting your manuscript to PLOS ONE. After careful consideration, we feel that it has merit but does not fully meet PLOS ONE’s publication criteria as it currently stands. Therefore, we invite you to submit a revised version of the manuscript that addresses the points raised during the review process.**

**Please note the both Reviewers suggested the same additional quality controls. Also, please proceed to review the minor points pointed out.**

**Please submit your revised manuscript by Feb 21 2025 11:59PM. If you will need more time than this to complete your revisions, please reply to this message or contact the journal office at plosone@plos.org. When you're ready to submit your revision, log on to** [**https://www.editorialmanager.com/pone/**](https://www.editorialmanager.com/pone/) **and select the 'Submissions Needing Revision' folder to locate your manuscript file.**

**Please include the following items when submitting your revised manuscript:**

- **A rebuttal letter that responds to each point raised by the academic editor and reviewer(s). You should upload this letter as a separate file labeled 'Response to Reviewers'.**
- **A marked-up copy of your manuscript that highlights changes made to the original version. You should upload this as a separate file labeled 'Revised Manuscript with Track Changes'.**
- **An unmarked version of your revised paper without tracked changes. You should upload this as a separate file labeled 'Manuscript'.**

**If you would like to make changes to your financial disclosure, please include your updated statement in your cover letter. Guidelines for resubmitting your figure files are available below the reviewer comments at the end of this letter.

If applicable, we recommend that you deposit your laboratory protocols in** [**protocols.io**](http://protocols.io/) **to enhance the reproducibility of your results. Protocols.io assigns your protocol its own identifier (DOI) so that it can be cited independently in the future. For instructions see:** [**https://journals.plos.org/plosone/s/submission-guidelines#loc-laboratory-protocols**](https://track.editorialmanager.com/CL0/https:%2F%2Fjournals.plos.org%2Fplosone%2Fs%2Fsubmission-guidelines%23loc-laboratory-protocols/1/010f0194403f8168-2bbe36ac-12fb-48a5-ad10-cca3b75ec9fa-000000/Z5SlDZTsHpZDTHHw2USC7OHOx4PKw8ECnxPNYYb-ME4=192)**. Additionally, PLOS ONE offers an option for publishing peer-reviewed Lab Protocol articles, which describe protocols hosted on** [**protocols.io**](http://protocols.io/)**. Read more information on sharing protocols at** [**https://plos.org/protocols?utm_medium=editorial-email&utm_source=authorletters&utm_campaign=protocols**](https://track.editorialmanager.com/CL0/https:%2F%2Fplos.org%2Fprotocols%3Futm_medium=editorial-email%26utm_source=authorletters%26utm_campaign=protocols/1/010f0194403f8168-2bbe36ac-12fb-48a5-ad10-cca3b75ec9fa-000000/RKDh2QYCTcGmAMSyYTTdMYhS2xSA0Lzbu-1B5kpMpcc=192)**.

We look forward to receiving your revised manuscript.**

**Kind regards,**

**Matteo De March**

**Academic Editor**

**PLOS ONE**

**-------------------------------------------------------------------------------------------------------**

**Journal Requirements:**

1. **When submitting your revision, we need you to address these additional requirements.

   Please ensure that your manuscript meets PLOS ONE's style requirements, including those for file naming. The PLOS ONE style templates can be found at**
   [**https://journals.plos.org/plosone/s/file?id=wjVg/PLOSOne_formatting_sample_main_body.pdf**](https://track.editorialmanager.com/CL0/https:%2F%2Fjournals.plos.org%2Fplosone%2Fs%2Ffile%3Fid=wjVg%2FPLOSOne_formatting_sample_main_body.pdf/1/010f0194403f8168-2bbe36ac-12fb-48a5-ad10-cca3b75ec9fa-000000/9OEW5g0j0U-LqJx4bhca40em0HL0bHjaIUyL8M0R7V8=192) **and**
   [**https://journals.plos.org/plosone/s/file?id=ba62/PLOSOne_formatting_sample_title_authors_affiliations.pdf**](https://track.editorialmanager.com/CL0/https:%2F%2Fjournals.plos.org%2Fplosone%2Fs%2Ffile%3Fid=ba62%2FPLOSOne_formatting_sample_title_authors_affiliations.pdf/1/010f0194403f8168-2bbe36ac-12fb-48a5-ad10-cca3b75ec9fa-000000/4eE44PbgqnVu-C8OhMRrUCoBXL1jcN8hFEyj63Aq6xI=192)

   **2. We note that the grant information you provided in the ‘Funding Information’ and ‘Financial Disclosure’ sections do not match.

   When you resubmit, please ensure that you provide the correct grant numbers for the awards you received for your study in the ‘Funding Information’ section.

   3. Thank you for stating the following financial disclosure:
   [The investigation presented here was funded by the Dirección General de Asuntos del Personal Académico (DGAPA) at the Universidad Nacional Autónoma de México (UNAM) through PAPIIT grants IN226523 and IG200223. This research utilized resources from the 16-ID LIX beamline of the National Synchrotron Light Source II, a user facility managed by the U.S. Department of Energy (DOE) Office of Science, which the Brookhaven National Laboratory operates under Contract No. DE-SC0012704. Arisbeth Guadalupe Almeida-Juarez was supported by a doctoral scholarship (2019-000002-01NACF-12588) granted by CONAHCyT, Mexico, and by the incentive as a member of the program Ayudantes de Investigador nivel III provided by the SNI. Enrique Rudiño-Piñera gratefully acknowledges financial support from the institutional budget from Instituto de Biotecnología , UNAM, and the economic incentive from Sistema Nacional de Investigadoras e Investigadores (SNI), México].**

   **Please state what role the funders took in the study. If the funders had no role, please state: ""The funders had no role in study design, data collection and analysis, decision to publish, or preparation of the manuscript.""
   If this statement is not correct you must amend it as needed.

   Please include this amended Role of Funder statement in your cover letter; we will change the online submission form on your behalf.**

**ANSWER to comment 3:** We examined the discrepancies between the "Funding Information" and the "Financial Disclosure" sections and confirmed that both are consistent with each other. Additionally, we have added the following sentence to the end of the new cover letter: “Finally, we declare that the funders had no role in the study design, data collection, data analysis, decision to publish, or preparation of the manuscript submitted to PLOS One.”

**4. Please note that in order to use the direct billing option the corresponding author must be affiliated with the chosen institute. Please either amend your manuscript to change the affiliation or corresponding author, or email us at plosone@plos.org with a request to remove this option**.

**ANSWER to comment 4:** We confirm that Professor Enrique Rudiño-Piñera (enrique.rudino@ibt.unam.mx), the corresponding author of this manuscript, is affiliated with the Instituto de Biotecnología at the National Autonomous University of Mexico, which has an active agreement with PLOSOne.

**5. When completing the data availability statement of the submission form, you indicated that you will make your data available on acceptance. We strongly recommend all authors decide on a data sharing plan before acceptance, as the process can be lengthy and hold up publication timelines. Please note that, though access restrictions are acceptable now, your entire data will need to be made freely accessible if your manuscript is accepted for publication. This policy applies to all data except where public deposition would breach compliance with the protocol approved by your research ethics board. If you are unable to adhere to our open data policy, please kindly revise your statement to explain your reasoning and we will seek the editor's input on an exemption. Please be assured that, once you have provided your new statement, the assessment of your exemption will not hold up the peer review process.**

**ANSWER to comment 5:** We have updated our original data availability statement to indicate that the data will be available upon acceptance. We are also uploading an Excel file labeled "RAW DATA," which contains the original data recorded for all the experiments described in the manuscript submitted to PLOSone.

**6. PLOS requires an ORCID iD for the corresponding author in Editorial Manager on papers submitted after December 6th, 2016. Please ensure that you have an ORCID iD and that it is validated in Editorial Manager. To do this, go to ‘Update my Information’ (in the upper left-hand corner of the main menu), and click on the Fetch/Validate link next to the ORCID field. This will take you to the ORCID site and allow you to create a new iD or authenticate a pre-existing iD in Editorial Manager.**

**Answer to comment 6:** Thank you and we are sorry for missing the required information. The ORCID ids for all authors are:

Enrique Rudiño- Piñera: [0000-0001-8170-6379](https://orcid.org/0000-0001-8170-6379)

Arisbeth Guadalupe Almeida-Juarez: 0000-0001-9963-9151

Liliana Pardo-López: 0000-0002-8927-1733

Shirish Chodankar: 0000-0003-4850-2926

Guadalupe Zavala: 0000-0001-8489-9823

**7. If any table files for review show as item type ‘other’ please change to item type ‘Table’ as the reviewer does not have access to these ’other’ files.**

**Answer to comment 7:** Thank you for the comment. We confirm that the only table submitted in this manuscript is not labeled as others.

**8. Please include captions for your Supporting Information files at the end of your manuscript, and update any in-text citations to match accordingly. Please see our Supporting Information guidelines for more information:** [**http://journals.plos.org/plosone/s/supporting-information**](http://track.editorialmanager.com/CL0/http:%2F%2Fjournals.plos.org%2Fplosone%2Fs%2Fsupporting-information/1/010f0194403f8168-2bbe36ac-12fb-48a5-ad10-cca3b75ec9fa-000000/nFaTpvI9AqF5MEy7DTvgMyfv7LzVHW_zLGT71ADhzbQ=192)**.**

**Answer to comment 8:** Thank you for the comment. We confirm that the figures in the supplementary material have captions.

[Note: HTML markup is below. Please do not edit.]

Reviewers' comments:

Reviewer's Responses to Questions

**Comments to the Author**

1. Is the manuscript technically sound, and do the data support the conclusions?

The manuscript must describe a technically sound piece of scientific research with data that supports the conclusions. Experiments must have been conducted rigorously, with appropriate controls, replication, and sample sizes. The conclusions must be drawn appropriately based on the data presented.

Reviewer #1: Yes

Reviewer #2: Yes

2. Has the statistical analysis been performed appropriately and rigorously?

Reviewer #1: N/A

Reviewer #2: Yes

3. Have the authors made all data underlying the findings in their manuscript fully available?

The [PLOS Data policy](http://track.editorialmanager.com/CL0/http:%2F%2Fwww.plosone.org%2Fstatic%2Fpolicies.action%23sharing/1/010f0194403f8168-2bbe36ac-12fb-48a5-ad10-cca3b75ec9fa-000000/6ulzEu92jB4YkPz-FDSnl8Y1P-OYOaFDXzy8HYhmtZ0=192) requires authors to make all data underlying the findings described in their manuscript fully available without restriction, with rare exception (please refer to the Data Availability Statement in the manuscript PDF file). The data should be provided as part of the manuscript or its supporting information, or deposited to a public repository. For example, in addition to summary statistics, the data points behind means, medians and variance measures should be available. If there are restrictions on publicly sharing data—e.g. participant privacy or use of data from a third party—those must be specified.

Reviewer #1: No

Reviewer #2: Yes

4. Is the manuscript presented in an intelligible fashion and written in standard English?

PLOS ONE does not copyedit accepted manuscripts, so the language in submitted articles must be clear, correct, and unambiguous. Any typographical or grammatical errors should be corrected at revision, so please note any specific errors here.

Reviewer #1: Yes

Reviewer #2: Yes

**5. Review Comments to the Author

Please use the space provided to explain your answers to the questions above. You may also include additional comments for the author, including concerns about dual publication, research ethics, or publication ethics. (Please upload your review as an attachment if it exceeds 20,000 characters)**

**Reviewer #1: The present study reports novel oligomeric forms of a catechol dioxygenase, an enzyme that is generally regarded as a dimeric protein.
Protein chemists are aware that proteins can form aggregates and aggregation is usually associated with (partial) unfolding, particle formation and loss of activity.

Here it is shown that before large aggregates form, a dimeric protein can form distinct tetramers and octamers, in addition to larger oligomers. The catechol dioxygenase tetramers retained full activity, demonstrating that an enzyme that retained activity after storage could nevertheless have been changed by the storage procedure. Additional quality control by light scattering or analytical gelfiltration would be advisable to control the storage procedure.

The present manuscript contains useful data, however the presentation of the results and the discussion should be improved. It remained unclear why the enzyme was lyophilized in the first place. The general problem of quality control of stored proteins should be discussed in more detail. The importance of the present study for bioremediation should also be outlined much more clearly.
Except for CD and PAGE, the results for the not-lyophilized enzyme are missing. Please add this data for gel filtration and SAXS.**

Thank you for your observation regarding the use of a lyophilized version of SfC12DO. To clarify, we have added the following lines in the revised manuscript:

Page 12, line 231-236: “In a preliminary characterization of SfC12DO oligomers, freshly purified SfC12DO was subjected to an initial SAXS (static mode) measurement. However, the samples exhibited signs of protein unfolding (S2 Fig.). During shipping to the synchrotron facility, the protein was unable to withstand the transport conditions and precipitated. To mitigate aggregation and enhance stability during shipping and long-term storage, we investigated lyophilization as a preservation strategy”.

Furthermore, regarding the information that was previously missing about the non-lyophilized SfC12DO, this data is now presented in the new Figure 4 and in Supplementary Figure S3.

We acknowledge that we did not provide any arguments regarding the protein stability or perform quality control on the lyophilized SfC12DO. The main reason for this omission is that our study focused on demonstrating that the enzyme, despite changing its quaternary structure, retains its enzymatic activity in the reconstituted sample. We agree that if our goal had been to prove the applicability of this lyophilized enzyme in bioremediation, additional experiments and controls would have been necessary. However, this objective is beyond the scope of the present study.

**Minor issues**

**1. Abstract, “All these characteristics make SfC12DO a very promising candidate for extensive bioremediation applications in polluted soils or waters. “ – why? please elaborate.**

**Answer to comment 1:** First, we acknowledge that our claim stating that SfC12DO is a “very promising candidate for extensive bioremediation applications” is based solely on its ability to maintain enzymatic activity in a reconstituted sample that was previously lyophilized. We agree that to support such a strong statement, additional experiments and controls must be conducted beyond the results presented in this work. Consequently, the following sections of the manuscript have been revised:

In the abstract the final new sentencein Page 3, line 38-39 is: “All these characteristics make SfC12DO a putative candidate for bioremediation applications in polluted soils or waters.”

Page 25, line 495-496: “...to solve problems like, potentially, bioremediation of soils and water”

Page 25, line 499: The sentence “and its potential implications in bioremediation” were erased.

**2. The authors state, “All relevant data are within the manuscript and its Supporting Information files.” , however I could not find any raw data. Please add excel or csv files with raw data. Add PDB files of the models as supplementary information.**

**Answer to comment 2:** You are correct; we did not include the raw data files in the original submission. Along with the corrected version, we are uploading an Excel file containing the raw data for figures 1 to 9 and supplementary figures S2 to S5, also protein models and SAXS data were added.

**3. Difference between SfsC12DO, Sf12DO and SfC12DO?**

**Answer to comment 3.** Thank you for your observation. There is no distinction between SfsC12DO, Sf12DO, and SfC12DO; these variations stem from an inconsistency in the use of acronyms in the original text. All terms refer to the same protein, SfC12DO, as clarified in the revised version of the manuscript. We apologize for the error.

**4. page 3 line 44, the β is missing**

**Answer to comment 4.** Thank you for pointing out this mistake; you are correct. We have corrected the text, and the missing "β" has been added on page 3, line 44 of the revised manuscript. We appreciate your attention to detail.

**5. Introduction: please add the alternative name 1,2-dihydroxybenzene for catechol**

**Answer to comment 5.** Thank you for your suggestion. We have updated the introduction to include the alternative name "1,2-dihydroxybenzene" for catechol, enhancing clarity and avoiding misinterpretations on page 3, line 45 of the revised manuscript.

**6. Merk → Merck**

**Answer to comment 6.** Thank you for identifying that error. We have corrected "Merk" to "Merck" in the revised version of the manuscript on page 6, line 112..

**7. correction of “sample types lyophilized-reconstituted”**

**Answer to comment 7:** Thank you for the observation. To clarify the samples used for each experiment, the following lines were modified in the revised manuscript:

Page 6, line 123-124: “SfC12DO samples were resuspended in degassed and filtered Buffer C (S1 Table) and the protein concentration was adjusted to 0.3 mg/ml, with a sample volume of 200 μl”

**8. ε = 16.8 mM, ε = 16.8 M, seems wrong and these are wrong units for an absorption coefficient (M-1 cm-1).**

**Answer to comment 8:** We have reviewed and revised the notation in the manuscript to use the correct units and values. The proper expression for the molar absorption coefficient is ε = 16.8 M⁻¹ cm⁻¹. We appreciate your attention to this detail. The correct use and units for absorption coefficient can be seen on page 7, line 135, and Figure 5 caption.

**9. Figure 2, secondary structure cannot be predicted with ESpript, this software just displays it.**

**Answer to comment 9:** Thank you for pointing this out. We have revised the text to accurately state that ESpript was used to display the secondary structure rather than predict it. This correction has been incorporated into the revised manuscript in the caption for Figure 2 as follows: **“ A)** The secondary structure of SfC12DO was visualized using ESpript 2.0. In this panel, *acc* represents the solvent accessibility index, and *hyd* stands for the hydrophobicity index “

**10. Figure 3, text font too small, peaks should be labelled p1, p2…, please include elution volumes of SfC12DO elution peaks in the inset (logMW vs. Ve/Vo). Please add a gelfiltration chromatogram of the enzyme which was not lyophilized for comparison. There isn’t a reference to Fig 2A in the main text, only 2B and 2C are referenced. 2C, RMSD compared to what?**

**Answer to comment 10:** To address your comments, we built a new version of Figure 3, named Figure 4, as you can see from the updated figures. The reference for Figure 2A was added to the revised version of the manuscript on page 12, line 239: “Secondary structure analysis of lyophilized and reconstituted SfC12DO was crucial to determine whether the protein remained properly folded. The predicted secondary structure of SfC12DO can be visualized (Fig 2A)”

To address your comment about the RMSD values, in the new Figure 2 caption, the following segment was added, page 13, line 265-266: “The root mean squared deviation (RMSD) is provided, comparing the calculated and experimental CD spectra

**11. Correction of “The CD analysis of SfC12DO's showed”**

**Answer to comment 11:** To address your comment, the following lines were modified as follows:

Page 12, line 241-243: “CD analysis of SfC12DO showed slight changes in the content of secondary structure elements between non-lyophilized and lyophilized SfC12DO”

**12. “non-lyophilized protein (15 ± 0.25 U/mg).” If the value is 15.00 then change to 15.00 ± 0.25 U/mg**

**Answer to comment 12:** Thank you for your suggestion. We have revised the text to reflect the value with appropriate precision, additionally, we corroborated the value and found a mistake, the correct number should be 12.44 +/- 0.61 instead of 15+/- 0.25. The corrected value can be seen on page 17, line 347-348: “non-lyophilized protein (12.44 ± 0.61 U/mg)”.

**13. Figure 5, It is unclear which panel shows which elution peak and what the diagrams actually show. If this are experimental data, where are the theoretical curves corresponding to the models? Please redo this figure and legend so that it becomes understandable for readers who are not familiar with the SAXS technique. SAXS data of the not-lyophilized enzyme would be needed.**

**Answer to comment 13:** To attend to your observations, a new Figure, numbered Figure 6 in the revised version of the manuscript, was produced. In the new Figure 6, we compare the experimental data with the modeled values using WAXSiS, as seen in panels A, B, and C. Additionally, we added panels A.1, B.1, and C.1 with the Guinier plots for each case. Regarding the SAXS data for the non-lyophilized SfC12DO, the new supplementary figure S2 was produced.

**14. Fig S3, what are the colors , what is shown here?**

**Answer to comment 14:** To address your observation regarding the colours code, S3 Figure has been separated into two different Supplementary figures: the new S4 Figure, which shows the distance distribution function P(r), and the new S5 Figure, which presents the dimensionless Kratky plot. The colors represent different oligomeric states: blue (p4) for the dimer, green (p3) for the tetramer, red (p2) for the octamer, and black (p1) for larger aggregates. The colour code was also added to each of the captions of the new supplementary Figures.

**15. Fig. 6, there is no panel D. Panel C, I do not understand what is shown here**

**at all.**

**Answer to comment 15:** Thank you for your observation, it was our mistake. To correct this, in the new Figure 7 captions, the error was corrected as follows:

 “C) Root Mean Square Deviation (RMSD) values show a comparison between *P. arvilla* C12DO (PDB: 2AZQ) and SfC12DO SAXS models for chains A and B. The results highlight structural differences between the models, emphasizing the chain-specific deviations measured”

**16. “In contrast, diffusion problems might prevent it” – it = the substrate?**

**Answer to comment 16:** Yes, "it" refers to the substrate. This clarification can be found on **page 22, line 426**.

**Reviewer #2: The authors describe the multimeric states of a catechol dehydrogenase enzyme and its impact on its activity. Overall, the manuscript is well laid out. I have a few recommendations for the authors and a few clarifications for the methods.**

**1. In Figure 2C, the authors have a table explaining the structural nuances for the few studied C12DOs; this would be improved for the reader if they could add the structures and their comparisons for domains and catalytic sites that would convey much more effectively.**

**Answer to comment 1:** The requested structural representations, including the structures of the studied C12DOs and their comparisons focusing on protein domains and catalytic sites, were already included in the new Figure 2C.

**2. What is the buffer for the enzymatic activity assay? Does the assay performance change for the Lyo and non-lyo material in different salinity?**

**Answer to comment 2**: The enzymatic activity assay was carried out using Buffer B, as stated in page 7, line 134 of the corrected manuscript. Furthermore, the assay was performed under identical salinity conditions for both lyophilized and non-lyophilized samples.

**3. For Figure 3B, can the authors add both the native and denaturing gel bands? Also, why not have all four peaks on the gel? They're tentatively in the right MW size range to run a native gel for all four peaks.**

**Answer to comment 3**: The native and denaturing gel bands have been added to the new Figure 3. However, not all four peaks are visible on the gel because the multimer of peak 1 exceeds the gel's size limit and does not run properly. You can see it represented as a band near the upper limit of the gel (Figure 3B, lane 4)

4. There definitely needs to be an overlay of the SEC profile for the non-lyo and lyo material to make that clear these multimers reflect the nature of the material.

**Answer to comment 4:** An overlay comparing the SEC profiles for both lyophilized and non-lyophilized materials have been added to the new Figure 4 as requested.

**5. IF there are gel lanes for all four peaks, then the enzymatic assay from those fractions is a good continuation.**

**Answer to comment 5:** Thank you for your observations. To address them the caption for the new Figure 3 changes as follows: “Fig 3. Oligomerization Analysis of the SfC12DO Protein: A) SDS-PAGE (12%) was performed on the purified SfC12DO to compare lyophilized and non-lyophilized samples. Lane 1 contains a molecular weight marker (kDa). Lane 2 shows the lyophilized SfC12DO, while Lane 3 displays the non-lyophilized SfC12DO. B) A semi-native PAGE (8%) was conducted to analyze the oligomeric distribution of SfC12DO. Lane 1 includes BSA as a molecular marker (kDa). Lanes 2 and 3 are two replicates of the non-lyophilized SfC12DO, and Lane 4 presents the lyophilized SfC12DO. The semi-native PAGE analysis of the lyophilized SfC12DO reveals various oligomeric species, including dimers, tetramers, hexamers, octamers, and high-ordered aggregates (greater than 20 subunits).”

Additionally, a new Figure 4 was constructed with the following caption: “**Fig 4. SEC elution profile of SfC12DO (lyophilized and non-lyophilized)** SfC12DO was eluted from a Sephacryl 200 HiPrep 16/60 column. Four distinct peaks were observed for SfC12DO lyophilized: peak 1 (p1) and peak 2 (p2) with undetermined molecular weights, peak 3 (p3) with an estimated M_W_ of ~113 kDa, and peak 4 (p4) with a M_W_ of ~77 kDa. The red dashed line represents the elution peak of non-lyophilized dimeric SfC12DO, also at M_W_ of ~77 kDa. On the right, the calibration curve standard is shown.”

After the update, the new Figure 5 has replaced the previous Figure 4 in the manuscript. With the information provided by the new Figures 4 and 5, we believe that the new Figure 5 is much easier to follow.

**I think the authors making these edits will improve the overall manuscript**

**6. PLOS authors have the option to publish the peer review history of their article (**[**what does this mean?**](https://track.editorialmanager.com/CL0/https:%2F%2Fjournals.plos.org%2Fplosone%2Fs%2Feditorial-and-peer-review-process%23loc-peer-review-history/1/010f0194403f8168-2bbe36ac-12fb-48a5-ad10-cca3b75ec9fa-000000/MLjYAKqLENUbYq8YcA9dKGQtk6x_OYUCPd6ahSt2KBA=192)**). If published, this will include your full peer review and any attached files.


If you choose “no”, your identity will remain anonymous but your review may still be made public.


Do you want your identity to be public for this peer review? For information about this choice, including consent withdrawal, please see our** [**Privacy Policy**](https://track.editorialmanager.com/CL0/https:%2F%2Fwww.plos.org%2Fprivacy-policy/1/010f0194403f8168-2bbe36ac-12fb-48a5-ad10-cca3b75ec9fa-000000/4BZ1r7SXHTgTfg61A9k8_GBE9ngspYrQSU0OGTpd0_4=192)**.**

**Reviewer #1: No**

**Reviewer #2: No**

**[NOTE: If reviewer comments were submitted as an attachment file, they will be attached to this email and accessible via the submission site. Please log into your account, locate the manuscript record, and check for the action link "View Attachments". If this link does not appear, there are no attachment files.]

While revising your submission, please upload your figure files to the Preflight Analysis and Conversion Engine (PACE) digital diagnostic tool,** [**https://pacev2.apexcovantage.com/**](https://track.editorialmanager.com/CL0/https:%2F%2Fpacev2.apexcovantage.com%2F/1/010f0194403f8168-2bbe36ac-12fb-48a5-ad10-cca3b75ec9fa-000000/huySv91YQmohkPVgwtRdVL051pfnSvLhtJ1VjLbOW2c=192)**. PACE helps ensure that figures meet PLOS requirements. To use PACE, you must first register as a user. Registration is free. Then, login and navigate to the UPLOAD tab, where you will find detailed instructions on how to use the tool. If you encounter any issues or have any questions when using PACE, please email PLOS at figures@plos.org. Please note that Supporting Information files do not need this step.**
